# The nanoparticle protein corona formed in human blood or human blood fractions

**DOI:** 10.1371/journal.pone.0175871

**Published:** 2017-04-17

**Authors:** Martin Lundqvist, Cecilia Augustsson, Malin Lilja, Kristoffer Lundkvist, Björn Dahlbäck, Sara Linse, Tommy Cedervall

**Affiliations:** 1 Center for Molecular Protein Science, Biochemistry, Lund University, Lund, Sweden; 2 Department of Translational medicine, Lund University, University Hospital SUS, Malmö, Sweden; 3 Upper 2^nd^ school, Klippan, Sweden; 4 NanoLund, Lund University, Lund, Sweden; National Institutes of Health, UNITED STATES

## Abstract

The protein corona formed around nanoparticles in protein-rich fluids plays an important role for nanoparticle biocompatibility, as found in several studies during the last decade. Biological fluids have complex compositions and the molecular components interact and function together in intricate networks. Therefore, the process to isolate blood or the preparation of blood derivatives may lead to differences in the composition of the identified protein corona around nanoparticles. Here, we show distinct differences in the protein corona formed in whole blood, whole blood with EDTA, plasma, or serum. Furthermore, the ratio between particle surface area to protein concentration influences the detected corona. We also show that the nanoparticle size *per se* influences the formed protein corona due to curvature effects. These results emphasize the need of investigating the formation and biological importance of the protein corona in the same environment as the nanoparticles are intended for or released into.

## Introduction

During the last decades, nanotechnology has emerged with new and possibly revolutionary applications in medicine, chemical industry, and in analytical and other technological setups [1–11]. Within the field of medicine, the development of nanoparticles for target-specific drug delivery has received particular attention. However, there is at least one major obstacle for successful target-specific drug delivery, i.e. interference from the biological macromolecule corona around the nanoparticle [12–17]. Nanoparticles may enter our body by different routes; inhalation, food intake, or intravenous and intracutaneous injections for medical applications are examples. However, regardless of the mode of entry, nanoparticles will be dispersed in biological fluids. In such environment, biomolecules will adsorb to the nanoparticle surface and form a corona. The stability and composition of the corona will vary dependent on nanoparticle size and surface chemistry. Moreover, the corona is not a static entity; it will exchange over time to adapt its composition to the surrounding biological environment [14]. Some particles will have a hard core of biological macromolecules interacting strongly with the nanoparticle surface, characterized by high affinity and often also low off-rates. The hard corona may be surrounded by an outer layer of weakly associated biological macromolecules characterized by lower affinity and higher off rates. Other kinds of nanoparticles will have a “soft” corona only, i.e. most of the biological macromolecules will be in rapid exchange with molecules in the surrounding environment [18]. However, regardless of whether the corona is “hard” or “soft”, the biological macromolecules that surround a nanoparticle will be important for its fate since this corona is what cells “see” and interact with [19].

A commonly investigated nanoparticle application is the development of target-specific drug carriers. Here, the corona may have an important role as plasma proteins have been shown to block ligands attached to the nanoparticle surface or compete with the interaction between ligands and cell receptors [20–22]. Dell’Orco *et al*. have previously modelled targeted delivery in the presence of an exchanging corona, to identify factors that are important for successful targeting [20, 21, 23]. The size of the bound proteins and their binding affinity for the particle surface were identified as very important. However, the particle size was found to be even more crutial. In the current work, this feature is addressed in experiments and confirmed to be important.

To the best of our knowledge, all studies characterizing the protein corona are performed in serum or plasma stabilized with different agents. In this work we investigate the corona composition as a function of the source of the plasma proteins, i.e. dependent on the treatment of blood after withdrawal. Blood can, after withdrawal, be treated in different ways to generate different liquids, conventionally named as: whole blood, whole blood with EDTA or Citrate, blood plasma or blood serum. We find that the protein corona composition differ between these four different biological fluids. As the protein corona is important for target delivery, this will have implications for the *in vitro* evaluation of a target-specific nanoparticle system.

We also show that the ratio between particle surface area and biological macromolecules in a sample affect the composition of the corona.

Knowledge about composition and dynamics of protein coronas is vital for the development of successful future nanoparticle-based medical applications. However, our current methods to analyze the corona may not be the optimal ones. In this paper, the limitations of the current methods are highlighted and the research fields is encouraged to find new suitable methods.

## Materials and methods

### Silica nanoparticles

Four different sized silica particles were kindly provided by AKZO NOBEL (www.colloidalsilica.com/eka.asp). The silica particles came in colloidal dispersions (basic pH ~11–13) of different concentrations, 30–50% w/v. The particle solutions were diluted, dialyzed and then the particle concentration was determined before use (see S1 File for details). The size and z-potential for the particles are reported in Table A in S1 File.

### Blood fraction and protein corona preparation

Blood was donated by one healthy individual (ML) at one occasion into two different vacutainer tubes, untreated tubes (which was used to prepare the whole blood samples and the serum samples) and EDTA treated tubes (which was used to prepare the EDTA stabilized whole blood samples and EDTA stabilized plasma samples).

The whole blood sample, in untreated tubes, was kept in constant agitation (to minimize the clot formation) from the withdrawal to the mixing with silica nanoparticles ca. 15 minutes after withdrawal. Other untreated tubes with freshly donated blood was kept still to allow the blood to clot. After that the remaining solution was decanted and centrifuged at 1.5 kRCF for 5 minutes and the supernatant was decanted and used as serum.

The blood in the EDTA treated tubes where either used directly, i.e. EDTA stabilized whole blood samples, or the tubes were centrifuged at 2 kRCF for 10 minutes and the supernatant was decanted and used as EDTA stabilized plasma. All experiments with these blood fractions were carried out by the donor (ML).

30 μL silica nanoparticles (or 15 μL of 9.5 nm particles), in 10 mM phosphate, pH 8.0, 0.15 M KCl, was mixed and incubated either with 150 μL whole blood, whole blood with EDTA, blood plasma or blood serum. This gives ~4 times more particle surface area in the sample for the 23, 13, and 9.5 nm particles compared to the 76 nm particle. The whole blood sample was incubated for 5 minutes while the other samples were incubated for 2 hours. The particles were pelleted by centrifugation (16 kRCF, 5 min), washed three times with 200 μl 10 mM phosphate, pH 7.4, 0.15 M NaCl, 1 mM EDTA and the vials changed after each washing step. Bound protein were removed from the particles by adding 2xSDS- loading buffer (10% w/v SDS, 20% w/v glycerol, 10% v/v 2-mercaptoethanol, 125 mM Tris-HCl, pH 6.8, bromophenol blue) and separated by 12% SDS-PAGE.

### Protein corona preparation for detection with mass-spectrometry

For these experiments and the thrombin generation assay (see further down) citrated plasma were used. The citrated plasma was prepared from a pool (to limit the traceability to a specific person) of blood from healthy anonymous human volunteers. The blood was withdrawn into citrate vacutainer tubes, and platelet-poor plasma was prepared by centrifugation at 2 kRCF at 25°C for 15 minutes twice and stored in aliquots at -80°C. Lund University Hospital ethic committee has approved all experiments in this article conducted with human plasma.

The protein coronas were investigated using mass-spectrometry (see further down) after preparation according to the previous section except that 15 μL 9.5 nm particles, or 60 μL 76 nm particles, were added to 1200 μL citrated plasma and 1 mL buffer was used in the washing steps.

### Protein identification by mass spectrometry

After the separation of proteins by SDS/PAGE (12%), bands were excised from the gel and identified as described [24]. Briefly, the gel-bands were reduced and alkylated, digested with trypsin and the resulting peptide mixtures were separated and analyzed with an Applied Biosystems 4700 MALDI TOF TOF instrument. Spectra were analyzed by MASCOT software to identify tryptic peptide sequences matched to the International Protein Index (IPI) database (www.ebi.ac.uk/IPI/IPIhelp.html). The mass spectrometry analysis were performed one time, however, the generation of the protein corona and detection of the corona with SDS-PAGE had been performed >3 times before the mass spectrometry analysis. Only proteins with Protein Score >65 were considered as hits.

### Thrombin generation assay

The silica particles were dialyzed against 10 mM HEPES, 150 mM NaCl, pH 8, for the rest according to the procedure stated in SI. Citrated plasma, platelet poor, was used for the thrombin generation assay. The amount of thrombin formed in plasma/NP samples were monitored using the thrombin generation assay as previously described [25] with the following modifications. Natural phospholipids (PL), 20/20/60 PS:PE:PC, were prepared. Sample mixture: 40 μL plasma + 40 μL 0.5 mg/mL for 76 nm (and the other silica particles were diluted to the same surface area/mL) were pre-incubated at 37°C for 15 min. 20 μL fluorogenic substrate (Z-Gly-Gly-Arg- AMC HCl) was added and coagulation was initiated by adding 20 μL PL/CaCl_2_ mixture. All reagents were diluted in HBSBSA (HBS buffer supplemented with 5 mg/mL BSA) and final concentrations were approximately 4.2 μM phospholipids, 300 μM fluorogenic substrate, and 16 mM CaCl_2_. Since no tissue factor is added, the activation by nanoparticles of the intrinsic pathway was studied. Three repeats were done for all experiments.

## Results and discussion

### Blood fraction preparation and protein corona purification

In a drug delivery application, the tagged nanoparticle will probably first encounter blood or interstitial fluid if delivered by intracutaneous or intravenous injection. Blood is a very complex fluid with red and white blood cells, platelets, and, among other biological macromolecules there are around 4000 different proteins or posttranslational modified proteins [26]. Blood can, after withdrawal, be treated in different ways to generate different liquids, conventionally named as follows

- Whole blood. Contains all blood components and will start to clot (the clotting can be delayed by gently agitating the solution).
- Whole blood with EDTA or citrate. Contains all blood components but will not clot as the cascade mechanism is inhibited due to chelation of calcium by EDTA or citrate.
- Blood plasma. Whole blood stabilized with EDTA or citrate. The platelets, red, and white blood cells have been removed after separation by gentle centrifugation.
- Blood serum. Contains what is left in solution after the clotting cascade has finished. Cells, platelets and blood clot have been separated by centrifugation.

Whole blood can also be stabilized with heparin. Heparin binds to and activates anti-thrombin, which in turn inactivates thrombin and other proteases involved in blood clotting [27]. Heparin-stabilized whole blood has not been used in this study.

The general procedure to purify nanoparticles with their layer of biological macromolecules [12, 13, 15] consist briefly of the following steps. 1. Nanoparticles are mixed with a biological fluid and incubated for different times to let the protein layer form around the nanoparticle and reach equilibrium with its surrounding. However, blood and other biological fluids will in physiological conditions start to degrade which may affect the corona. We have showed theoretically that for a co-polymer particle that the protein corona reached equilibrium within four hours after mixing, however, the change in the corona were limited between 1,5–4 hour [23]. The incubation time has to be a compromise, taking into account the system that is investigated and the goal of the specific study. 2. The formed nanoparticle-biological macromolecule complex is separated by centrifugation and the pellet, containing the complex, is washed repeatedly. Proteins and other substances bound to the particles in the pellet belong to the hard corona since all weakly associated biological molecules have been washed away. 3. The bound proteins are dissociated from the particles by incubation in SDS loading buffer (10% w/v SDS) and identified by mass spectrometry after separation by SDS PAGE.

### Centrifugation and detected protein corona

In most published studies of the protein corona around nanoparticles, centrifugation is used to isolate the protein:particle complex. However, this method to separate the interesting object, the complex, from the bulk has some severe drawbacks. We have previously pointed out the effect of different ratios between particle surface and protein concentrations on the amount of detectable pellet after centrifugation [15, 28]. Furthermore, gradually decreasing the available surface area showed that in human blood proteins the detected proteins in the corona on silica particles was dependent on the ratio of proteins and particle surface area [29] and in an excess of proteins, the corona was dominated by a single protein. A general problem with the centrifugation technique is that the pellet is analyzed. However, the formed pellet will depend on several experimental parameters of which some are difficult to control.

Firstly, centrifugation time and speed needed to pellet particles will depend on particle size, density, and on proteins that are bound to the particles. Spherical particles, which are in stable colloidal suspensions, will due to differences of their density have different pelleting times at a certain applied RCF. Table B in S1 File shows some calculated pelleting times for particles with similar density as silica, polystyrene [30] and gold particles of size 10 and 100 nm. After 3 min centrifugation at 20 kRCF, only the 100 nm gold particles would be close to 100% removed from the bulk. All other particles require longer centrifugation times. The situation will be more complex when a layer of proteins are added to each particle. Protein densities, average ~1,37 g/cm^3^ [31–33], are different from the densities of the nanoparticles they interact with, and the density of the nanoparticle-protein complex will be a weighted average of the two densities. However, with a density of ~1,37 g/cm^3^, one or a couple of layers of proteins at a particle surface will only speed up the sedimentation for one of the three theoretical particles shown in Table B in S1 File. Proteins are still used to stabilize nanoparticles in colloidal solution, i.e. proteins help keeping nanoparticles in solution (for example BSA is used to stabilize gold nanoparticles), relying on repulsive forces between the proteins. However, attractive or depletion forces may dominate and proteins can sometimes drive aggregation of the particles, which will again change the size and density of the particle protein complexes. It is therefore important to experimentally determine the needed centrifugation time and speed to completely pellet the nanoparticle-protein complexes. This is rarely done and, as evident from Table B in S1 File, sometimes unrealistic centrifugation times will be needed. The difficulties with centrifugation techniques highlight the need to use more than one separation technique to study the protein corona.

Secondly, formation of large nanoparticle-protein aggregates can trap proteins that does not adsorb to the nanoparticle surface, i.e. they are not part of the corona. Sometimes large pellets are reported after 3 min centrifugation of samples in which 5–50 nm nanoparticles are mixed with protein. This should in most cases not be possible, see Table B in S1 File. When nanoparticles are mixed with a biological solution *in vitro*, a high concentration of nanoparticles is added to a fixed volume of biological sample. This may explain the pelleting because at the mixing time point, aggregates with nanoparticles and proteins are formed due to depletion forces [34] or protein driven aggregation of nanoparticle-protein complexes and these are pelleted by centrifugation. In the aggregation process, molecules may be trapped inside the formed aggregates, as schematically illustrated in Figure D in S1 File. If the particle surface area is large, low affinity proteins may bind that would not bind in situations with a large excess of proteins and these interactions will be stabilized inside the aggregate. Furthermore, the amount of available surface area also affects protein-driven aggregation of the nanoparticles [35, 36]. The trapped proteins may be released in the analysis of the complex and therefore contribute to the detected protein corona without being a part of it. Aggregation may partly explain coronas with numerous proteins detected. Examples with a large number of detected proteins, can be found in our own work Lundqvist *et al*. [14, 15] where coronas for 6 different polystyrene particles are shown, in contrast to coronas with relative few proteins, for example from our own work Cedervall *et al*. [13] with two different co-polymer particles and Ferreira et al. [37] with one mannan particle.

Unfortunately, since we do not have a working alternative, the work presented in this article is done with the centrifugation technique.

### Protein coronas formed in different blood derivatives

**Fig 1:**
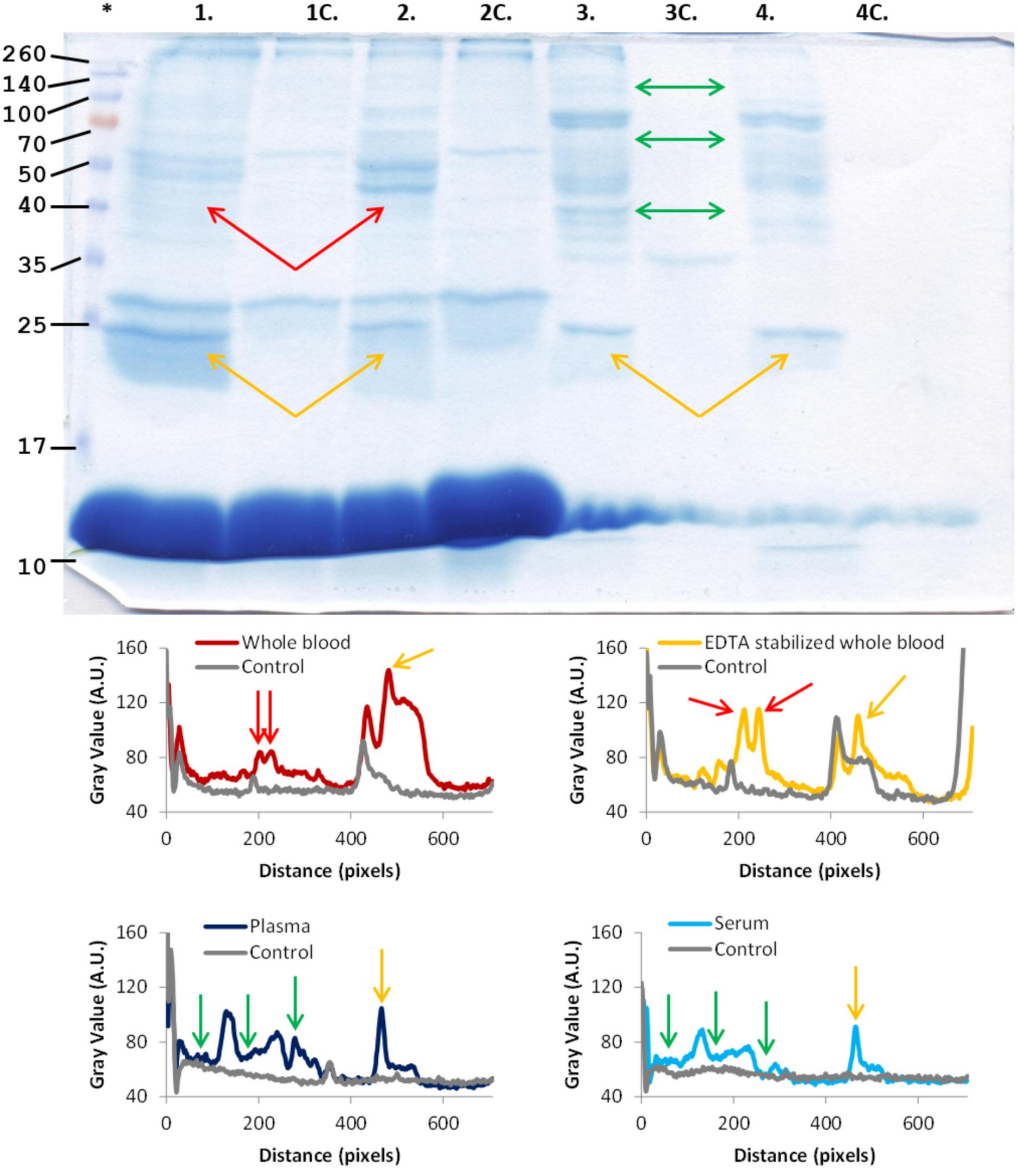
Protein coronas, formed around 76 nm silica particles in whole blood and different blood derivatives. Top panel: protein corona formed in (1) whole blood (2) EDTA stabilized whole blood (3) EDTA stabilized blood plasma and (4) blood serum. The respective blood fractions are shown in the control lanes (1c, 2c, 3c and 4c) The * marks the lane with Mw standards with M_w_ in kDa. Bottom panel: comparison of the intensity of gel bands after band intensity analysis [41, 42] for coronas and controls. The arrows high-light some of the differences found between different blood derivatives.

The set up and performance of experiments with unstabilized whole blood requires extra care. If the blood is not stabilized, with EDTA, citrate or heparin, the blood clotting mechanism starts immediately after the blood has been extracted. The clotting cascade can be reduced/delayed by gentle rocking of the blood. This means that any experiment with unstabilized whole blood should start as soon as possible after the blood donation. In this study, one of the authors donated the blood and carried out all the experiments with that blood. The blood was transferred, under constant agitation, from the clinic to the lab by the author (~10 min). At the lab the experiment with the untreated whole blood was initiated immediately. To further avoid blood clotting, untreated whole blood was only incubated 5 minutes with the NPs, under constant rocking, before the purification of the protein corona started. However, even with this set up the blood clotting interfere with the corona determination as shown in Fig 1 and Figure A in S1 File. A longer incubation time may increase the risk that NPs, together with their protein corona, will be incorporated into the clot. The blood plasma and blood serum were prepared from the donated blood. The EDTA stabilized whole blood is ready to use directly after donation.

To determine the composition of the protein corona formed in the different blood derivatives, silica nanoparticles, 76 nm, were mixed with whole blood, whole blood with EDTA, and with EDTA-stabilized blood plasma and serum. After separation by centrifugation the bound proteins were desorbed by 10% w/v SDS in loading buffer and the proteins in the corona separated by 1D SDS-PAGE and visualized by coomassie staining.

Fig 1 shows that there are clear differences in the coronas formed in whole blood without or with EDTA. The red arrows highlight two bands around 50 kDa. These protein bands are more pronounced on nanoparticles in whole blood with EDTA than in whole blood. These bands mainly represent fibrinogen β and γ. Fibrinogen will, at the end of the coagulation cascade, form a fibrin clot. The reason less fibrinogen is seen in whole blood may be that partial clotting has occurred in that sample. The fibrin clot will migrate as a very large protein complex which may be seen at the top of the gel as there are more high molecular protein bands in the whole blood than in EDTA-stabilized blood. The protein corona formed in plasma is likewise different, see the green arrows, from the protein corona formed in serum. Finally, there are clear differences between the protein corona formed in whole blood compared to the protein corona formed in plasma or serum. Although the detected protein coronas are different in the four blood derivatives, they have one very pronounced band, pointed out by the yellow arrows, in common. This band represents apolipoprotein A-I, the main structural and functional protein in the cholesterol carrier HDL (High Density Lipoprotein Particles). Apolipoprotein A-I is found in most nanoparticle coronas, no matter nanoparticle size or material [12, 38–40].

There are numerous proteins in the two whole blood sample controls, presumably due to the presence of platelets and red and white blood cells in these samples, see Figure A in S1 File. During the washing steps, the cells will break and the intracellular proteins will be released into the washing buffer. The released protein may form a secondary corona. However, it is clear that despite the background shown in the controls, specific corona proteins can be seen in the nanoparticle samples. At the bottom of the gels there are very strong protein bands representing hemoglobin, which most likely is a result of pelleted red blood cells. There are fewer proteins in the controls for the blood plasma and blood serum samples compared to whole blood, supporting the conclusion that the background in whole blood derives from the cells.

**Fig 2:**
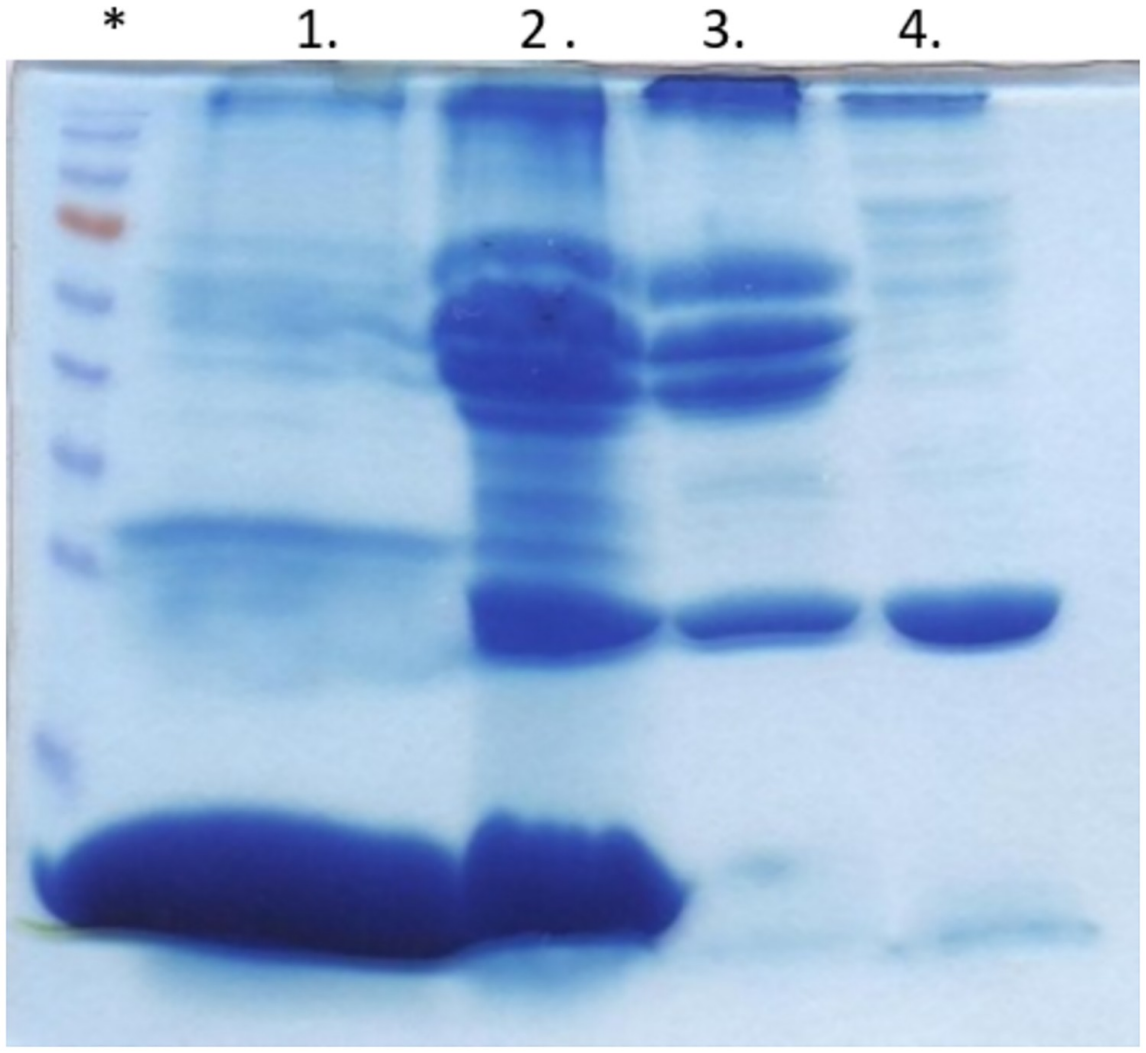
Protein coronas, formed around 9.5 nm silica particles in different blood derivatives. Top panel: 1 = protein corona from whole blood, 2 EDTA stabilized whole blood, 3 blood plasma, and 4 blood serum. The * marks the Mw standard.

**Fig 3:**
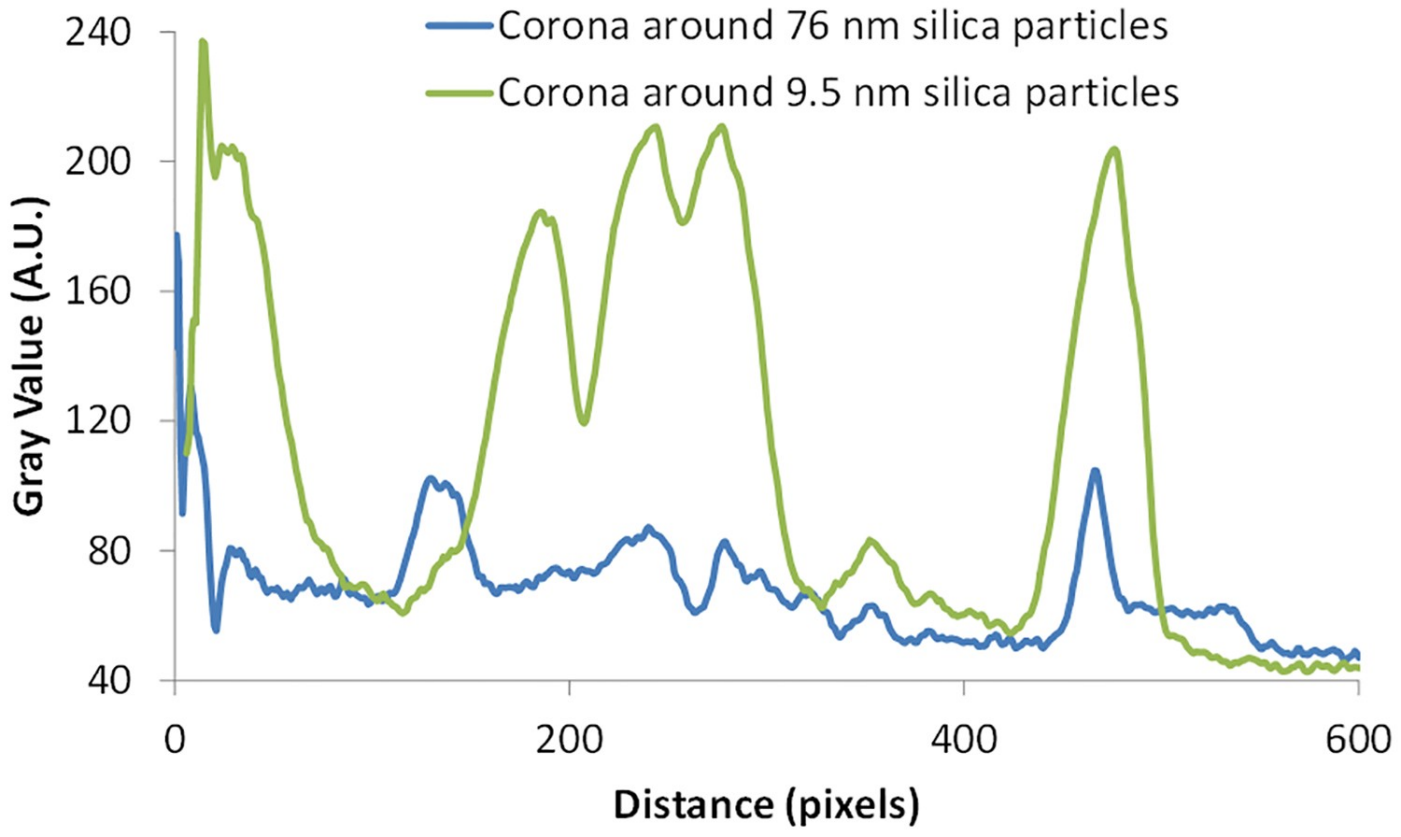
Comparing the intensity of different bands for two plasma corona from silica nanoparticles. Plasma corona from 9.5 nm silica particles in green and from 76 nm silica particles in blue. The x-axis has been adjusted for the 10 nm silica sample since the data comes from different gels.

It has previously been shown that the size matters as the composition of the formed protein corona differs between particles with identical material and surface modification but of different size. This is especially true when particles in the range 3–50 nm are compared to particles over 100 nm in diameter [15, 16]. Therefore, smaller (9.5 nm) silica particles were also analyzed in the four different blood solutions, the experiment setup resulted in a difference of a factor of ~4 times more particle surface area in the samples compared to the 76 nm particles. The result is shown in Fig 2. In a similar manner as for the 76 nm silica particles, there are clear differences in the formed coronas for the 9.5 nm particles. An overlay of the formed protein corona on the 9.5 and 76 nm silica particles in plasma, shown in Fig 3, reveals that particle size is important for the corona composition. Another striking difference is the lack of a pronounced Apolipoprotein A-I band for the 9.5 nm silica particles in whole blood.

The particle size influence on the formed protein corona was further explored by determining the formed protein corona around 13 and 23 nm silica particles. For both particles the surface area was the same as for the 9.5 nm particles, i.e. ~4 times more than the 76 nm particles. The gel in Figure B in S1 File shows the detected coronas around 13 and 23 nm silica nanoparticles. The detected coronas for the whole blood samples resembles more the 76 nm sample, with a pronounced apolipoprotein A-I band, than the 9.5 nm sample (even though they are much closer in size to the 9.5 nm particle). In serum, the detected coronas can be seen as a transition from the 9.5 to the 76 nm nanoparticle corona, with a decrease of the amount of detected apolipoprotein A-I with increasing size of the particle. This is probably an effect of the change in curvature/size of the nanoparticles. However, the really interesting data are the coronas for the whole blood with EDTA and plasma samples. The amount of detected protein from whole blood with EDTA for the 13 and 23 nm particles is lower than for the 9.5 nm particles, indicating a relatively sharp cutoff for maximum apolipoprotein A-I binding. There are also clear differences between the coronas for the 13 and 23 nm particles, see arrows in Figure B in S1 File.

Even more striking is the almost empty lanes for the plasma samples. The plasma samples for the 13 and 23 nm samples generated small pellets that decreased further in the washing steps. The experiment was repeated, except for the whole blood samples, and the results are shown in Figure C in S1 File. In general, the detected coronas in Figure C in S1 File resemble the coronas shown in Figs 1, 2 and Figure B in S1 File. However, in Figure C in S1 File two samples pellets, for the 13 and 23 nm silica particles in plasma, were pooled and run on the gel which results in some detected proteins (i.e. they contain double amount of sample compared to Figure B in S1 File. The formed coronas around the 13 and 23 nm particles in plasma, in the current experimental setup, stabilizes the nanoparticles in the solution and hinder the formation of large nanoparticles-protein aggregates.

The results in Figs 1–3 and Figure B-C in S1 File show that the formed protein corona around a nanoparticle is highly dependent of the composition of the biological solution. This can affect *in vitro* evaluation of the efficiency of a drug carrier candidate and should be taken in consideration while testing nanoparticles.

### Proteins in the protein corona

The next two experiments, the MS detection and blood coagulation test, were conducted with citrate stabilized plasma. The blood was donated by several, anonymous to the authors, individuals. The individual blood plasma fractions had been pooled before delivered to the authors.

**Fig 4:**
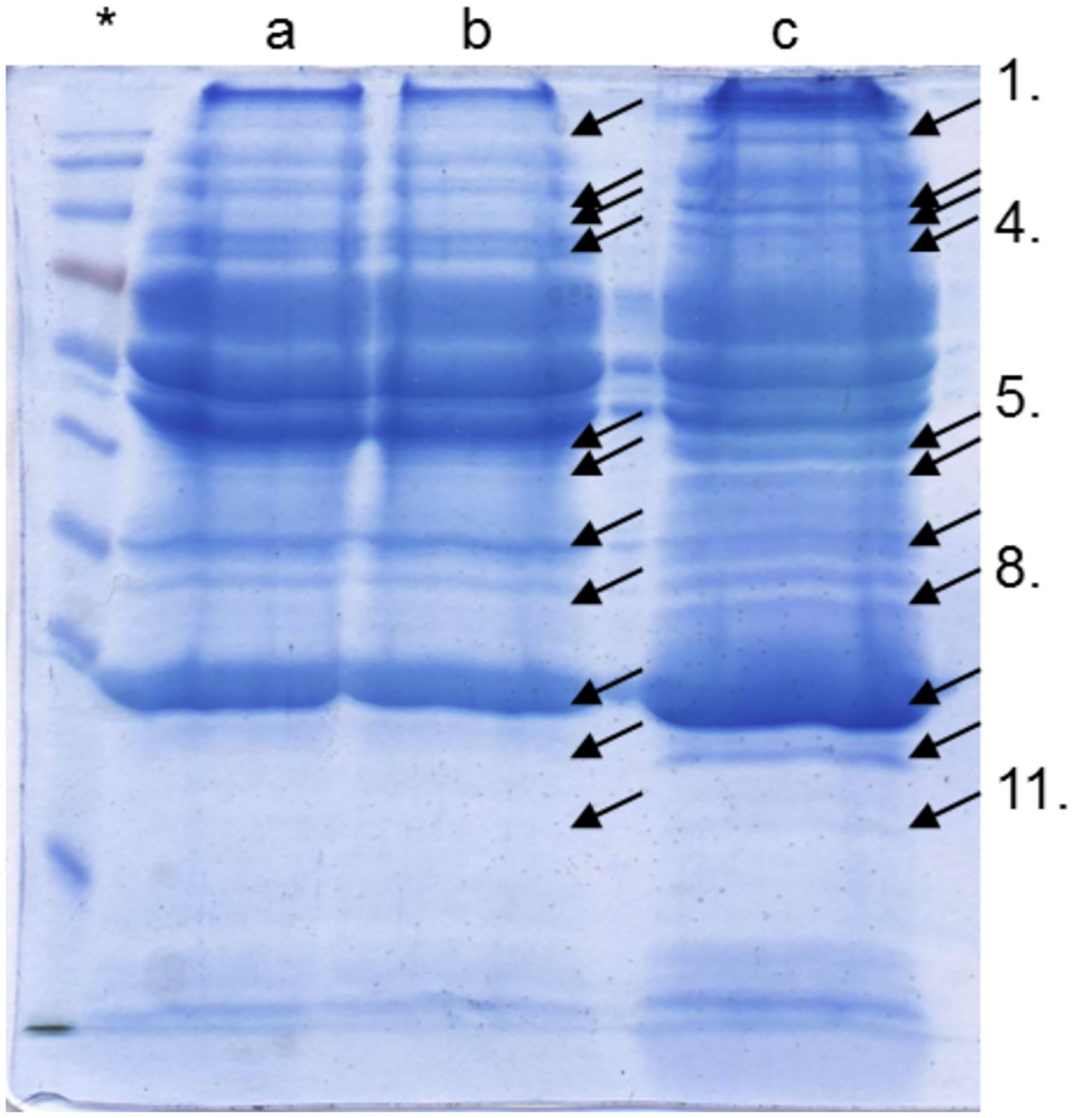
Protein corona SDS-gel bands analyzed with mass spectrometry. Lanes a and b: the plasma protein corona around 76 nm silica particles. Lane c: the plasma protein corona formed around 9.5 nm silica particles. Arrows indicate areas that were excised for analysis by limited proteolysis and the results are listed in Table 1. * is the molecule weight standard.

MS was used to further explore the difference between the coronas around the 9.5 and 76 nm particles. Unlike previous experiments citrated plasma was used instead of EDTA stabilized plasma. The plasma was mixed with silica particles as before, but with ~4 times more plasma volume to the same amount of NPs. Finally, two individual pellets were pooled at the first washing step for the 9.5 nm sample. Comparison between the SDS-gels in Figs 2 and 4 reveals that the corona compositions differ between the different experiments. Hence, the ratio between blood and investigated particle surface area matters for the protein corona composition as previously have been reported for plasma and polystyrene and silica particles [15, 29] and for nasal lavage fluid and iron oxide wielding particles [28].

Areas from the gel shown in Fig 4 were excised and analyzed by limited proteolysis using trypsin. The areas were selected to represent differences between the coronas i.e. a reasonably strong band for one of the particles and no or a weak band for the other particle. The band likely representing apolipoprotein A-I was confirmed in both coronas. Identified proteins for the two coronas are presented in Table A and C in S1 File.

As Table 1 shows, the two coronas differ. In the 76 nm silica particle is detected, among others, coagulation factor XII, while for the 9.5 nm silica particles, among others, complement factor H and complement C1q subcomponent subunit B are detected. These proteins are part of two different biological processes, the coagulation cascade and the complement system. The data indicate that the two differently sized silica particles may affect humans differently.

**Table 1:** Identified proteins.

		9,5 nm Silica particles		76 nm Silica particles
^a^	A.No^b^	Protein Name	A.No^b^	Protein Name
1	P08603	Complement factor H		n.d.^c^
2	P00747	Plasminogen	P00748	Coagulation factor XII
3	P06396	Gelsolin	P04196	Histidine-rich glycoprotein
4		n.d.^c^	P04196	Histidine-rich glycoprotein
5	P27169	Serum paraoxonase/arylesterase 1	P02671	Fibrinogen alpha chain
	P02679	Fibrinogen gamma chain		
	P02671	Fibrinogen alpha chain		
6	Q03591	Complement factor H-related protein 1	P04196	Histidine-rich glycoprotein
7		n.d.^c^	P02649	Apolipoprotein E
8	P02746	Complement C1q subcomponent subunit B	P02649	Apolipoprotein E
			P02671	Fibrinogen alpha chain
9	P02647	Apolipoprotein A-I	P02647	Apolipoprotein A-I
10	P02671	Fibrinogen alpha chain		n.d.^c^
11	P02671	Fibrinogen alpha chain		n.d.^c^

^a^ Cut part from gel as indicated in Fig 4.

^b^ UniProt Accession Number.

^c^ No protein detected in this band.

### Silica nanoparticles and blood coagulation

**Fig 5:**
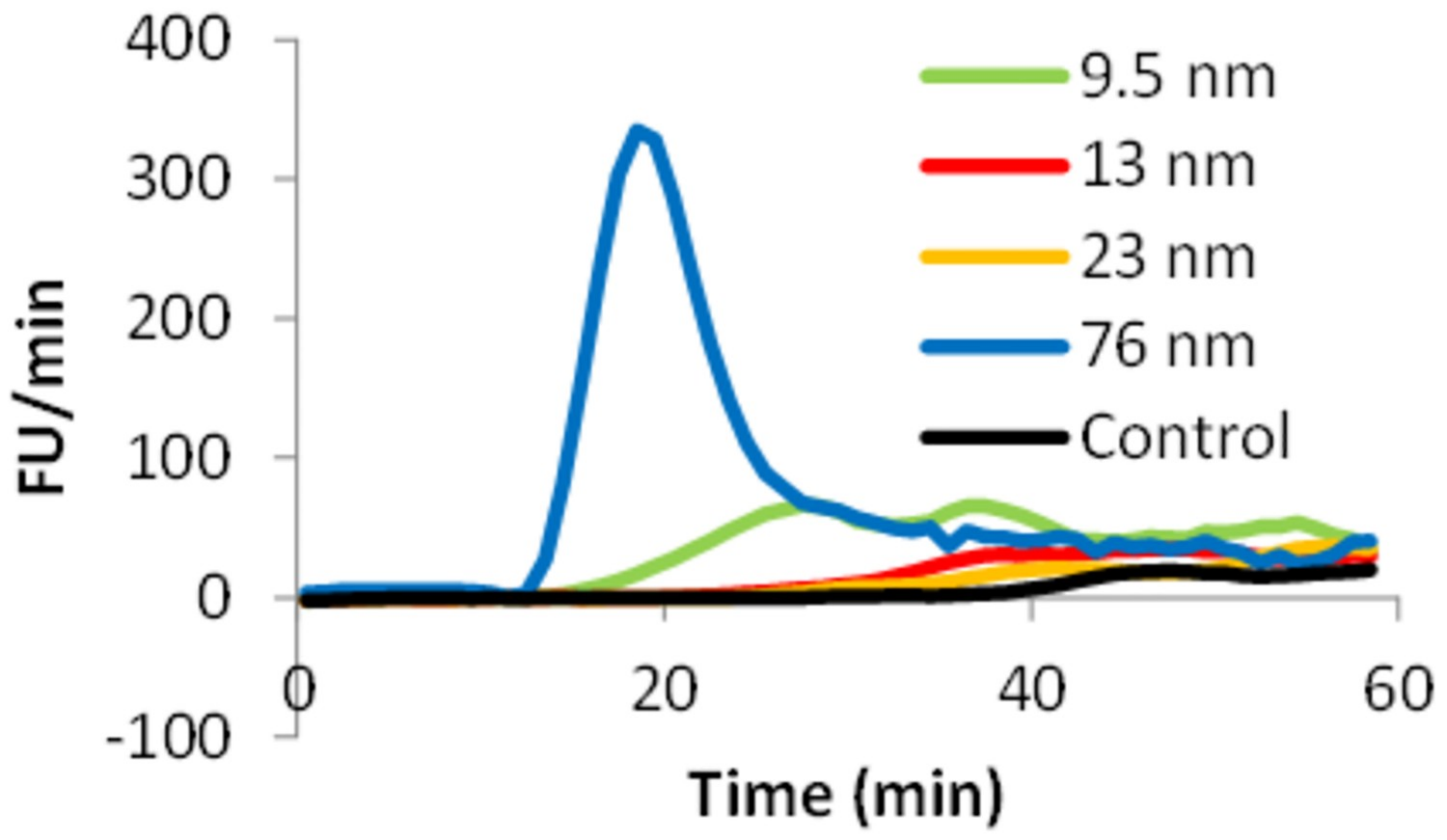
Nanoparticle induced thrombin generation. Black line) 9.5 nm particles, dashed black line) 13 nm particles, gray line) 23 nm particles, dashed gray line) 76 nm particles and dotted black line) control. The first derivative, fluorescence units/min, is shown (means of n = 3).

We have previously shown that both size and surface modification of polystyrene nanoparticles are important factors for how they affect the coagulation cascade [43, 44]. Similar experiments were conducted for the silica nanoparticles of four different sizes. The results are shown in Fig 5 (raw data in Figure E in S1 File). Interestingly, the different silica particles generate quite different responses in the modified thrombin-generation assay. The 76 nm nanoparticles were able to act as a surface for activation of the intrinsic pathway of blood coagulation in plasma, while the 13 and 23 nm particles are similar to the control and the 9.5 nm particles are somewhere between. These findings support the difference shown for the corona compositions for the four particles in plasma in Figs 1, 2, Figure B-C in S1 File.

## Conclusions

The simple experiments presented here show the complexity of determining exactly the fate of a nanoparticle after entering a biological fluid. Even when whole blood is investigated; the system is still a simplification from the *in vivo* situation in which, among other things, there may be additional effects by pressure and flow.

The detected protein corona around a nanoparticle will depend on, among other things: the composition of the biological solution, the particle size, and the ratio between biological solution and particle surface area.

The centrifugation method may not be the optimal method to use to isolate nanoparticle-biological macromolecule complexes for analysis. Alternative methods to study protein corona around nanoparticles in biological fluids need to be developed in order to obtain a more realistic protein composition of the corona.

## Supporting information

S1 File Contains Table A: DLS data for silica particles, Table B: Example of theoretical sedimentation times for particles traveling a distance of 1 cm at 20 kRCF, Table C: Detected proteins and there protein score, Figure A: Pictures showing the pellets after centrifugation of whole blood samples with nanoparticles, Figure B: Protein coronas, formed around 13 and 23 nm silica particles in different blood derivatives, Figure C: Repeats of experiment with blood derivates, Figure D: Schematic illustration of the formation of particle—protein complex with the entrapment of proteins within the complex, and Figure E: Raw data for the thrombin generation experiment. The file also includes detailed description of preparation of the nanoparticle stock solutions.
(DOCX)
