# Supplementary material for: The nanoparticle protein corona formed in human blood or human blood fractions
Source: PLoS One. 2017 Apr 17;12(4):e0175871. doi: 10.1371/journal.pone.0175871 (PMC5393619; doi:10.1371/journal.pone.0175871)
Supplement: S1 File — Contains Table A: DLS data for silica particles, Table B: Example of theoretical sedimentation times for particles traveling a distance of 1 cm at 20 kRCF, Table C: Detected proteins and there protein score, Figure A: Pictures showing the pellets after centrifugation of whole blood samples with nanoparticles, Figure B: Protein coronas, formed around 13 and 23 nm silica particles in different blood derivatives, Figure C: Repeats of experiment with blood derivates, Figure D: Schematic illustration of the formation of particle—protein complex with the entrapment of proteins within the complex, and Figure E: Raw data for the thrombin generation experiment. The file also includes detailed description of preparation of the nanoparticle stock solutions. (DOCX) [file pone.0175871.s001.docx]

**Supporting Information**

**Table A. DLS data for silica particles.**^1^

| Diameter^2^  (nm) | ^3^ | PdI^4^ | Zeta Potential (mV) |
| --- | --- | --- | --- |
| 9.5 | 9.5 | 0,139 | -22 |
| 13.2 | 13 | 0,174 | -22 |
| 23.3 | 23 | 0,054 | -36 |
| 76.2 | 76 | 0,055 | -43 |

^1^ All values are for particles in 10 mM phosphate buffer, 0.15 M KCl with pH 8.0.

^2^ Calculated according to number-weighted averages.

^3^ Name used to refer to the particle in the main text.

^4^ Polydispersity Index.

**Preparation of the nanoparticle stock solutions**

The particle solutions were diluted 5-10 times (in 10 mM phosphate buffer, 0.15 M KCl with pH 8.0) depending on their original concentration (30-50% w/v) to a concentration of ~6% w/v. Thereafter, the particles were extensively dialyzed, using 3500 MWCO tubing, against the same buffer, at 4°C. To be able to determine the concentration of the dialyzed solutions, 5 aliquots were taking from each dialyzed particle solution (5 aliquots of the buffer were also taken) and the water was evaporated in a heating block. The pellet weight (minus the pellet weight for the buffer controls) divided by the volume of the aliquot gave the concentration in % w/v. The concentrations were adjusted to 4% (w/v) with buffer.

The determined mass concentration together with the size determined with DLS (see S1 Table) was used to calculate particle surface area in the samples.


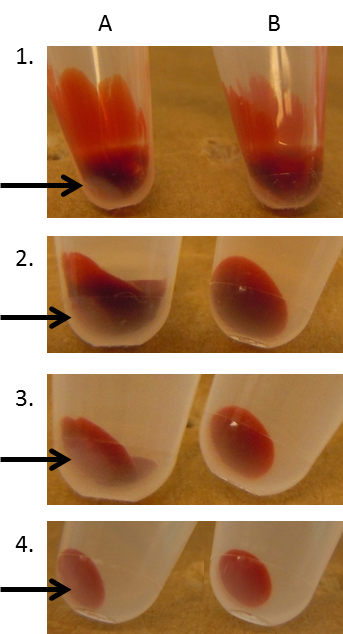


**Figure A. Pictures showing the pellets after centrifugation of whole blood samples with nanoparticles.** A = 76 nm silica sample and B = control sample. 1 = after fist centrifugation, 2 = after first wash step, 3= after second wash step and 4 = after third wash step. The arrows indicate the silica particle pellet.


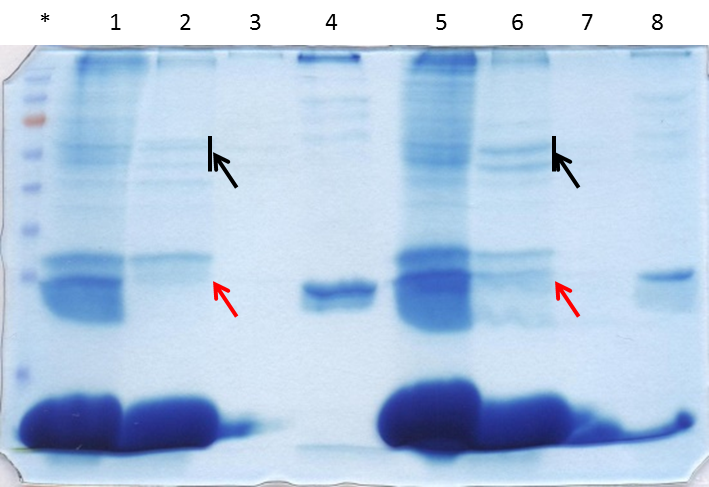


**Figure B. Protein coronas, formed around 13 and 23 nm silica particles in different blood derivatives.** Gel showing: * Mw standard, 13 nm silica particles with whole blood (1), whole blood EDTA (2) plasma (3) and serum (4) and 23 nm silica particles with whole blood (5), whole blood EDTA (6) plasma (7) and serum (8). Control samples are shown in Fig 1 of the main text. The arrows points out differences in the whole blood with EDTA samples for the 13 and 23 nm silica particles.


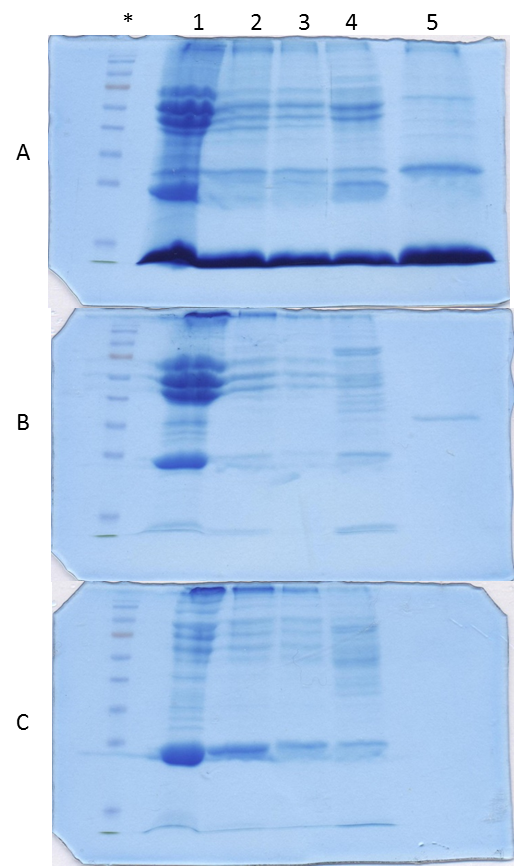


**Figure C. Repeats of experiment with blood derivates.** Gel A = whole blood EDTA, gel B = plasma and gel C = serum. Lane marked with * = Mw standard, 1 = 9.5, 2 = 13 nm, 3 = 23 nm and 4 = 76 nm silica particles. Lane marked 5 is the control sample. For the 13 and 23 nm samples the amount of starting material was doubled compared to the experiments reported in S2 Fig.


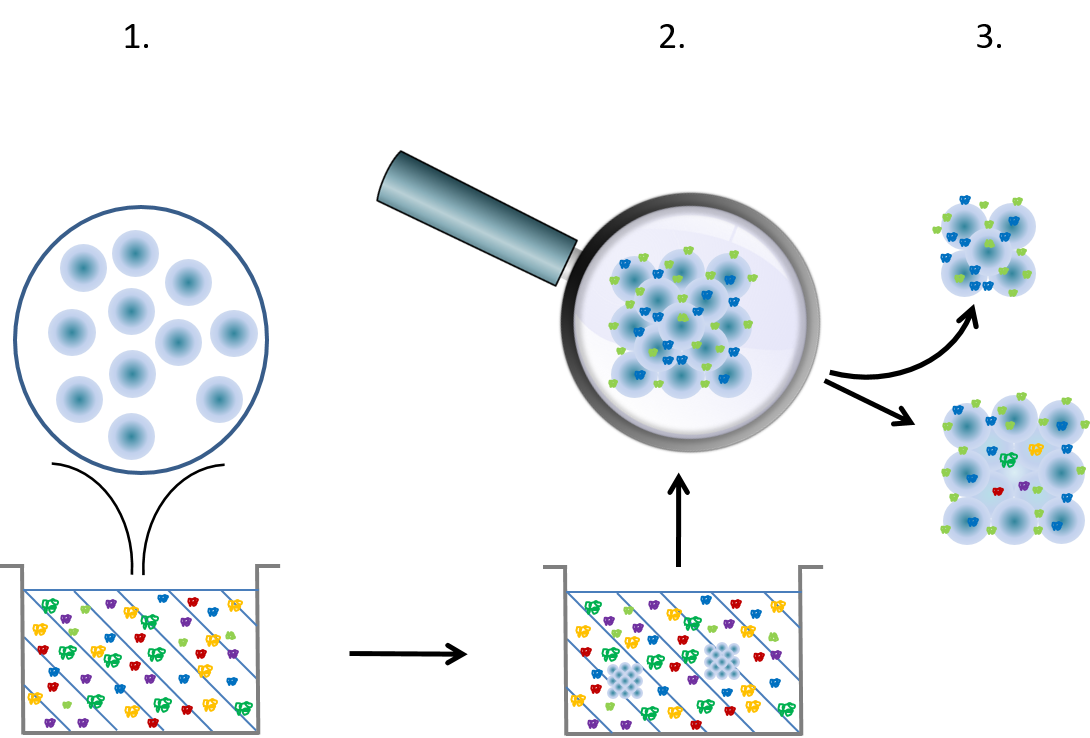


**Figure D. Schematic illustration of the formation of particle – protein complex with the entrapment of proteins within the complex.** Two processes can lead to the formation of particle aggregates; depletion and protein interaction driven aggregation. Both processes can lead to proteins entrapped, in solution, inside the formed aggregate. 1. Nanoparticles are added to a protein solution. 2. The light green and blue proteins adsorbs to the nanoparticles leading to the formation of particle-protein aggregates. 3. The particle-protein aggregate is opened to show a void in the aggregate containing unadsorbed but trapped proteins (red, yellow, green and purple) in solution.


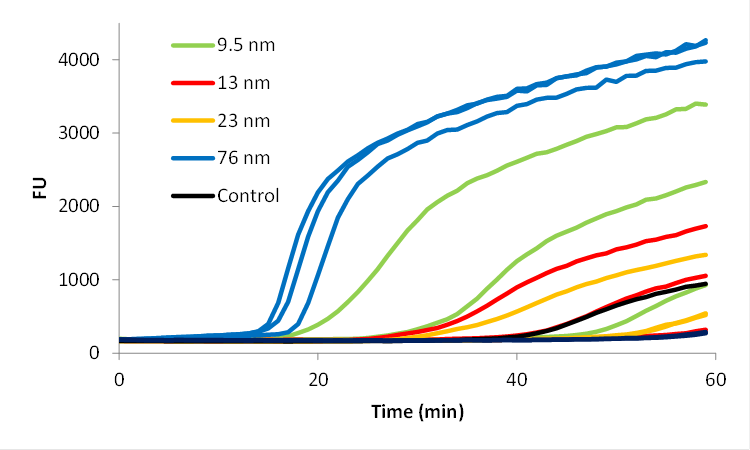


**Figure E. Raw data for the thrombin generation experiment.** Green) 9.5 nm particles, red) 13 nm particles, orange) 23 nm particles, blue) 76 nm particles and black) control. Three replicates of each sample.

**Table B. Example of theoretical sedimentation times for particles traveling a distance of 1 cm at 20 kRCF.**

|  | Density | Particle Size | Time for sedimentation^B^ |
| --- | --- | --- | --- |
|  | (kg/m^3^) | (nm) | (hour:minut:second) |
| Polystyrene | 1053 | 10 | 472:38:29 |
| Polystyrene | 1053 | 100 | 04:43:35 |
| Silica | 1900-2600^A^ | 10 | 25:03:00 |
| Silica | 1900-2600^A^ | 100 | 00:15:02 |
| Gold | 19320 | 10 | 01:22:02 |
| Gold | 19320 | 100 | 00:00:49 |

^A^ Different values can be found in the literature.

^B^ Calculated for a constant centrifugal force of 20 kRCF and a depth of solution of 1 cm according to the equation:

$$T=\frac{\left( 9l\eta\right)}{(2r^{2} \left( \rho_{p}- \rho_{s} \right)RCF)}$$

where: *T* = time in seconds

*l* = length

*η* = viscosity of solution, water at 20 °C = 1.002x10^-3^ Pa. S

*r* = radius of the particle

$\rho_{p}$ = density of the particle

$\rho_{s}$ = density of the solution, 1000 kg/m^3^ for water

$RCF$ = relative centrifugal force

**Table C. Detected proteins and their protein score.**

| Accession Number | Protein Name | Protein Score |
| --- | --- | --- |
| P08603 | Complement factor H | 84.4 |
| P00747 | Plasminogen | 92.5 |
| P06396 | Gelsolin | 76.9 |
| P27169 | Serum paraoxonase/arylesterase 1 | 73.9 |
| P02679 | Fibrinogen gamma chain | 73.6 |
| P02671 | Fibrinogen alpha chain | 72.8 |
| Q03591 | Complement factor H-related protein 1 | 100.0 |
| P02746 | Complement C1q subcomponent subunit B | 173 |
| P02647 | Apolipoprotein A-I | 437 |
| P02671 | Fibrinogen alpha chain | 262 |
| P02671 | Fibrinogen alpha chain | 168 |
|  |  |  |
| P00748 | Coagulation factor XII | 69.7 |
| P04196 | Histidine-rich glycoprotein | 509 |
| P04196 | Histidine-rich glycoprotein | 314 |
| P02671 | Fibrinogen alpha chain | 158 |
| P04196 | Histidine-rich glycoprotein | 171 |
| P02649 | Apolipoprotein E | 325 |
| P02649 | Apolipoprotein E | 81.5 |
| P02671 | Fibrinogen alpha chain | 67 |
| P02647 | Apolipoprotein A-I | 515 |
